# Pulse Oximetry Screening for Critical Congenital Heart Defects: A Life-Saving Test for All Newborn Babies

**DOI:** 10.3390/ijns5010014

**Published:** 2019-02-12

**Authors:** Andrew K. Ewer

**Affiliations:** 1 Institute of Metabolism and Systems Research, University of Birmingham, Birmingham B15 2TT, UK; a.k.ewer@bham.ac.uk; 2 Neonatal Unit, Birmingham Women’s and Children’s Hospital NHS Foundation Trust, Birmingham B15 2TG, UK

Congenital heart defects (CHD) are the commonest congenital malformations and remain a major cause of neonatal mortality and morbidity in the developed world [1,2]. Critical congenital heart defects (CCHD) are the most serious form of CHD, with an incidence of between two and three per 1000 live births [3]. Babies with CCHD are at risk of cardiovascular collapse, acidosis, and death in the first few days of life, usually following closure of the ductus arteriosus; therefore, early diagnosis is essential to reduce the possibility of these complications and also to improve outcomes following cardiac surgery [1,2].

In high-income countries, most babies are routinely screened for CCHD using antenatal ultrasound scanning and postnatal physical examination. However, both of these procedures have a relatively low detection rate, and up to a third of babies with CCHD may be discharged from hospital before a diagnosis is made [4,5].

Pulse oximetry (PO) measures blood oxygen saturations and is a well-established, accurate, non-invasive method of detecting low oxygen levels (hypoxaemia) [1,2]. The rationale for using PO to screen for CCHD is that hypoxaemia is present in the majority of cases of CCHD, but the degree of desaturation is often comparatively mild and may be clinically undetectable, even by experienced clinicians [6]. Therefore, the addition of PO screening (POS) following delivery will detect those babies with hypoxaemia, who can then be assessed and the presence of a CCHD established before the babies develop acute collapse [1].

Proof of concept and feasibility of POS was first established by a number of small single centre studies in the early 2000s, but the low prevalence of CCHD in these studies meant that there was insufficient evidence to precisely define the test accuracy, and firm recommendations for routine screening could not be made [2,7]. Between 2008 and 2012, several large European studies provided sufficient, robust evidence of test accuracy which could reliably inform the possible introduction of routine POS [8,9,10,11,12]. 

In 2012, a systematic review and meta-analysis of all available evidence (including nearly 230,000 screened babies) concluded that pulse oximetry screening was a moderately sensitive, highly specific test for detection of CCHD, which met the criteria for universal screening [13]. In 2014, the world’s largest POS study involving over 120,000 babies from China [14] demonstrated similar findings which essentially removed any remaining uncertainties about the performance of PO screening [15].

The addition of POS reduces the ‘diagnostic gap’ for CCHD [10] (i.e. those babies who are not detected by existing screening methods), and when POS is combined with antenatal ultrasound scanning and the newborn physical examination, between 92% and 96% of babies with CCHD are identified in a timely manner [16].

Attitudes towards POS are changing, and acceptance of the potential benefits is becoming more widespread. In 2012, a Lancet editorial commented ‘…surely, the question now is not “why should pulse oximetry screening be introduced?” but “why should such screening not be introduced more widely?”’ [17]. The papers within this special edition book for the *International Journal of Neonatal Screening* address many of the broader aspects of POS beyond the basic test accuracy; topics include acceptability, cost-effectiveness, screening in different settings—such as outside the newborn nursery environment and at altitude—and, importantly, implementation of POS in different countries and clinical settings and establishing a screening system which suits the local population.

Giving appropriate information and assessing the acceptability of any screening test—both to the patients involved and to the clinical staff who administer it—is vital if it is to be successful. Previous studies have shown that POS is acceptable to parents and clinical staff [12,18,19] and also that it does not appear to create additional anxiety in the mothers (including those who have a false positive result) [18]. In this special edition, Cloete et al. report feedback from parents on both the information they received prior to testing and their overall satisfaction of POS during a pilot screening study in New Zealand [20]. The cultural diversity and the mainly midwifery-led maternity system in this country make the positive responses received particularly pertinent. As part of their extensive overview of the implementation process of POS in the USA—the first country to legislate for mandatory POS of all newborns—Wandler and Martin also report on their systematic and highly effective approach to addressing the issues raised by such a huge undertaking [21].

As well as being acceptable, a new screening test must also be shown to be cost-effective. A number of previous studies in different countries have tried to address this issue [9,22,23,24].

In their review of this work in this special edition, Scott Grosse and colleagues provide a comprehensive analysis of the available evidence, including a revised estimate of cost (based on recent improved survival figures from the US following the introduction of POS), estimating that the cost could be as low as USD 12,000 per life-year gained [25].

Switzerland, Ireland, and Poland were the first countries to recommend routine POS [16,26], and in 2011, as described above, the USA was the first country to mandate this test [27,28].

Over the past five years, an increasing number of countries, including Canada [29], the Nordic countries [30], Saudi Arabia, Abu Dhabi, and Sri Lanka [26], have recommended routine screening. In Europe, significant progress has been made by a multinational group of clinicians working towards a Europe-wide implementation of PO screening [31] and recently publishing a European consensus statement, endorsed by leading figures from European Paediatric and Neonatal Societies [32]. In this special edition, further national recommendations are published from Germany [33] and South and Central America [34], in addition to a local study from Valencia, Spain [35], which was one of the precursors to the recent Spanish recommendation [36]. In the UK, almost half of all maternity units are screening [37] but there is no national recommendation as yet. In Australia, a different approach has been taken; in this special edition, Martin Kluckow suggests that pulse oximetry should be considered a ‘routine vital sign’ of general neonatal wellbeing rather than a test for a specific target such as CCHD [38]. This allows individual hospitals to screen in a manner which suits them and might make the process simpler and potentially more acceptable; this is an approach which seems to have worked in Australia, but in response, Gentles et al. argue that a structured national recommendation would ensure a more equitable service to the whole population [39]. Kluckow’s reply highlights the fact that national recommendations are often rather slow and cumbersome and that babies may miss out on screening until the recommendation is sanctioned [40].

Screening pathways (or algorithms) for POS within the published studies are variable [7,13,41,42]. The main differences are i) site of saturation measurement (the use of a single [post-ductal] saturation measurement or measuring both pre- and post-ductal saturations) and ii) the timing of screening (before or after 24 h of age).

Screening algorithms which use only a single post-ductal measurement are potentially quicker and easier, but investigation of the data from these studies and those using both pre- and post-ductal saturations show that a small number of babies with CCHD may be missed by using only a single post-ductal measurement [16,41,42]. With large populations, this number may become significant, and the benefits of using two measurements may outweigh the potential minor disadvantages [16,40,42]. Most of the recent recommendations advocate dual site measurements, but Riede and colleagues recommend post-ductal saturation measurement only [33]. An interesting alternative strategy is proposed by Walsh and Ballweg from Tennessee USA, who advocate post-ductal saturations with a higher threshold (97%) for the initial screen and then pre- and post-ductal saturations for those who require a repeat [43].

As with any screening test, it is important to consider the number of false positives (those babies who have a positive test but do not have CCHD), and the timing of the PO screen affects the number of false positive screens [13,41]. Later screening (>24 hours) has a lower rate of false positive tests [13,41]; however, between 30% and 80% of false positive babies have a significant respiratory or infective condition or non-critical CHD [16,25,42,44]. Earlier screening is mandatory in countries (such as the UK where the majority of babies are discharged within 24 h after birth or in the Netherlands where many babies are born outside of the hospital environment). In addition, screening after 24 h of age may result in up to half of babies with CCHD presenting before POS occurs, sometimes with an acute deterioration [16,42]. These factors must be considered carefully; although a lower number of false positives is advantageous in a screening test, if the majority of false positives have a serious non-cardiac condition which requires urgent treatment, this is clearly a significant additional benefit [19]. In addition, later screening—after 24 hours—may lead to more babies with CCHD becoming seriously unwell before testing takes place, which defeats the purpose of screening [16,42]. As more countries engage with POS and high-quality outcome data are collected, the nuances of modifying and refining the screening algorithms can be modelled with greater precision [42].

Screening babies born outside of the nursery e.g., at home, in a midwifery-led birthing centre, or on the neonatal intensive care unit (NICU), present particular challenges; with homebirth midwifery staff often leave the mother and baby shortly after birth, which means that POS must take place within a couple of hours. However, screening babies in this situation has been shown to be both feasible and acceptable in a small UK study [45] and a much larger Dutch study [46,47]. In this special edition, Narayen and colleagues present their experience screening such babies in the Netherlands [48] and Kim et al. report their findings of screening newborns on the NICU at moderate altitude (1700m) [49].

In summary, PO screening is feasible, cost-effective, and acceptable, and it also reduces the diagnostic gap for CCHD. This special edition of the *International Journal of Neonatal Screening* focuses on a number of issues which are entirely relevant to those who might be considering introducing such screening.

A universal programme of PO screening in newborns will increase the detection of CCHD, and importantly, it has also been shown to be useful in identifying other potentially life-threatening clinical conditions (such as respiratory problems and sepsis), which is an important additional advantage. In a very important report from the USA, Abouk et al. report a 33% reduction in mortality from CCHD in US states that had introduced POS compared with those where introduction had not yet occurred [50].

When defining the most appropriate screening algorithm, a balance must be struck between detection of a serious illness and limiting false positive results, and local circumstances may play a role in this respect. More data from larger populations may help to refine further the screening algorithm. Finally, it is also important to remember that PO screening is not a perfect test, and babies with CCHD may still be missed [15,16]. Therefore, PO screening should be used as an addition to existing screening methods, and health care workers and parents need to be aware of the limitations of the test.
